# Clinical and preclinical approach in AGA treatment: a review of current and new therapies in the regenerative field

**DOI:** 10.1186/s13287-024-03801-5

**Published:** 2024-08-15

**Authors:** Lorena Pozo-Pérez, Pilar Tornero-Esteban, Eduardo López-Bran

**Affiliations:** 1 Dermatology Department, Clínico San Carlos Hospital, Madrid, Spain; 2 Institute for Health Research of Clinico San Carlos Hospital (IdISSC), Madrid, Spain; 3 Cellular GMP Manufacturing Facility, Institute for Health Research of Clinico San Carlos Hospital (IdISSC), Madrid, Spain

**Keywords:** Androgenetic alopecia, Hair growth, Cell therapy, Stem cells, Conditioned medium, Bioinks, Nanomaterials

## Abstract

Androgenetic alopecia (AGA) is the most prevalent type of hair loss. Its morbility is mainly psychological although an increased incidence in melanoma has also been observed in affected subjects. Current drug based therapies and physical treatments are either unsuccessful in the long term or have relevant side effects that limit their application. Therefore, a new therapeutic approach is needed to promote regenerative enhancement alternatives. These treatment options, focused on the cellular niche restoration, could be the solution to the impact of dihydrotestosterone in the hair follicle microenvironment. In this context emerging regenerative therapies such as Platelet-rich plasma or Platelet-rich fibrine as well as hair follicle stem cells and mesenchymal stem cell based therapies and their derivatives (conditioned medium CM or exoxomes) are highlighting in the evolving landscape of hair restoration. Nanotechnology is also leading the way in AGA treatment through the design of bioinks and nanobiomaterials whose structures are being configuring in a huge range of cases by means of 3D bioprinting. Due to the increasing number and the rapid creation of new advanced therapies alternatives in the AGA field, an extended review of the current state of art is needed. In addition this review provides a general insight in current and emerging AGA therapies which is intented to be a guidance for researchers highlighting the cutting edge treatments which are recently gaining ground.

## Introduction

AGA is a dynamic and progressive hair loss disorder which affects men and women around the world. The incidence of AGA increases with age, affecting 80% of Caucasian men population and 30–50% bellow the age of 50 years old [1, 2]. A similar prevalence is observed in elderly women [3]**.** Although AGA is often considered a minor dermatological condition, hair loss has a huge impact on self-esteem and quality of life, hence its frequent association with anxiety and depression [4].

The etiology of AGA entails an intricate interplay among different genetic and hormonal factors, resulting in the miniaturization of hair follicles and alterations in the dynamics of the hair growth cycle, specifically the shortening of the anagen phase. AGA hormonal etiology is caused by DHT, an androgen derived from testosterone by 5-alpha reductase enzyme action. This hormone has a higher affinity towards androgen receptors (ARs) in the hair follicles. In fact, individuals with AGA have an overexpressed AR gene compared to controls [5, 6]. This situation leads to follicle miniaturisation after the expression of senescence genes [6, 7].

AR locus is located in the X cromosome hence it shows a X-linked inheritance. In addition several AR polimorfisms are known to be linked to a higher probability to suffer AGA [8]. Although 5α-reductase enzime is also a key factor in AGA development, SRD5A1 and SRD5A2 (5-αreductase genes) association studies do not showed any relation between them and AGA [9].

Although male AGA etiology is well known to be caused by DHT action in hair follicles, female AGA is related to a huge range of trigger factors, hence observed clinical differences between both sexes. For example women have a diffuse hair loss patron whereas all men keep their hair density in occipital areas. Female AGA cases are in many cases linked to hirsutism patients and in menopause period [10, 11] Estrogens exerts a protective impact probably due to their capacity to participate in androgen metabolism in the dermal papilla cells (DPCs).

Hair cycle includes three phases: hair growth phase (anagen) [12], regression phase (catagen) [13] and relative rest phase (telogen) [14]. In AGA patients, a shortening of the anagen phase is observed, so that the telogen phase sets in progressively. Hair becomes thinner and eventually the anagen phase turns so short that hair is not long enough to reach the skin surface [15] Testosterone and DHT act on the ARs in the DPCs by negatively modulating growth factors genes transcription and positively growth factor suppressors such as Transforming Growth Factor Beta (TGF-β) and Dickkopf-1 (DKK-1), both inducers of the catagen phase [16, 17]. It is well known that anagen phase is characterised by the proliferation of follicular cells, mainly epithelial cells and DPCs. The latter, together with bulge stem cells (BSCs), are the two main types of hair follicle stem cells involved in hair growth. Their importance has been documented detecting changes in their functionality in AGA patients [18, 19]. Inflammation is also one of the pathophysiological characteristics of AGA, as evidenced by the lymphocytes and mast cells infiltration around the bulge area [20–22].

## Material and methods

A systematic clinical trials review was conducted in ClinicalTrials.gov (https://clinicaltrials.gov/). The keywords “AGA” as Condition or disease and “CELL THERAPY” were used. A literature search about preclinical studies was conducted using Pubmed (https://pubmed.ncbi.nlm.nih.gov/) and introducing various combinations of the following terms: FINASTERIDE, AGA, CELL THERAPY, ASCs (Adipose derived Stem Cells), ASCs-CM (ASCs Conditioned Medium), MESOTHERAPY, PRP, MINOXIDIL, KETOKENAZOL, BICALUTAMIDE, CORTEXOLONE 17A-PROPIONATE, LASER THERAPY, HAIR TRANSPLANT, MURINE MODEL, MICE, MICRONEEDLING, WNT PATHWAY, JAK-STAT PATHWAY, MSCs and SVF (Stromal Vascular Fraction), REGENERATIVE THERAPIES, NANOPARTICLES, BIOINKS, GREEN NANOMATERIALS, HERBAL EXTRACTS, PHYTOMEDICINE amd 3D BIOPRINTING. The electronic databases were systematically searched until May, 2024.

## Conventional therapies

### Antiandrogens

Finasteride 1 mg/day is the only one oral FDA and EMA approved drugs for AGA treatment. This drug is a potent and specific inhibitor of the 5-alpha reductase (type II) which shows benefitial effects stopping hair loss in the 80% of the patients after one year-treatment [23]. Its primary action, which involves halting DHT production is responsible of adverse effects such as reduced eyaculation volume, loss of libido and erectile dysfunction. Furthermore, although prolonged treatments with Finasteride do not produce drastic semen alterations in healthy men, it may impact patients experiencing infertility symptoms, leading to controversial prescription in young men [24]. In general Finasteride levels are very low in semen hence its use is not restricted [25]. Moreover, Finasteride is also contraindicated in women due to its teratogenic effects during pregnancy and its potential risk of developing breast cancer [26].

Thus, efforts have been led to develop various topical formulations of Finasteride to mitigate systemic side effects associated with oral administration, such as sprays or nano-transferosomal gels. Studies indicate that topical Finasteride increases hair count, is well tolerated and is as effective as oral Finasteride [27–30].

Nevertheless, despite its effectiveness and minimal adverse effects, further research is essential to evaluate the long-term efficacy of hair regrowth, therapeutic safety, cost-effectiveness, patient tolerability and satisfaction with topical Finasteride among individuals with androgenetic alopecia. Although oral is the only Finateride formulation approved for AGA treatment in Europe, also recommmended as concomitant therapy after follicular unit transplantation procedure, topical solution is expected to be approved proximately due to the numerous ongoing and completed phase III clinical trials (NCT03004469/EUDRACT2015-002877–40).

Concomitantly Ketoconazole, a topical antifungal shampoo for the treatment of seborrhoeic dermatitis can be applied. Its anti-inflammatory and anti-androgenic properties by affecting steroid genesis enhance Finasteride effect by decreasing DHT levels in the male scalp [31, 32]. An improvement in the progression of AGA and hirsutism conditions in women was also observed [33].

On the other hand, Dutasteride, another selective inhibitor of both type-1 and type-2 5-alpha reductase enzymes was assessed as Dutasteride 0,5 mg showing superior outcomes in terms of hair density and hair width compared to Finasteride 1 mg treatment [34]. However, its clinical use remains limited as it is only approved in Mexico and Korea [26]. Currently its topical formulation is being tested in Europe (EUDRACT2022-001802–23).

A topical application of these inhibitor drugs is expected to be approved proximately if clinical trials continue showing as effective results as oral ones.

Other drugs that avoid AR activation are its antagonists such as Cortexolone 17a-propionate, Spironolactone or Cyproterone. In general these drugs do not achieve better results than approved ones (oral Finasteride and topical Minoxidil) and cause several adverse effects**.**

### Minoxidil

As aforementioned, Minoxidil is currently the only topical drug approved for AGA. Its application for hair growth was discovered as an adverse effect of hypertension treatment due to its vasodilator action by opening potassium channels. Its oral formulation is not prescribed for AGA because it promotes a systemic arterial hypotension caused by vascular smooth muscle relaxation. Its topical application is completely safe and it was approved with the aim of acting only on the scalp vessels. It increases blood flow thus an extra nutrients and oxygen supply to the hair follicle. Moreover, one study found cytoprotective activity resulting from the activation of prostaglandin synthase-1, the main isoform in DPCs [35]. Doses range from 2 to 5%, and like other hair growth stimulators, Minoxidil treatment can cause telogen follicles to fall out and be replaced by new ones [36]. Nevertheless, the efficacy in the population is still low, not exceeding 40% of treated patients after 24 weeks of treatment [23].

In order to improve Minoxidil outcomes several oral doses and sulphate compositions are being tested. Recently, a clinical study of 30 male participants has shown efficacy and safety of 5 mg daily dose of oral Minoxidil after being administered to patients for 24 weeks [37]. Lower oral doses such as 1.25 mg/day for 24 weeks, daily capsules containing minoxidil 0.25 mg and spironolactone 25 mg as well as a dose range of 0.25–2.5 mg in female hair loss also showed a clinical improvement and a hair shedding reduction in AGA patients [38–40] as well as the sublingual daily dose of 0.45 mg, tested in female and male AGA subjects with an acceptable safety and efficacy profile [41]. The most common adverse effects are irritant and allergic contact dermatitis on the scalp and facial hypertrichosis [26].

Throughout the years, several studies have pointed out the importance of sulphate as an effective supplement to Minoxidil treatment. In particular Minoxidil sulphate is known to be fourteen times more potent than Minoxidil tested in vitro follicles [42]. These results have been supported over the years. In 2019, Maekawa et al. reported hair growth promoting effects in an in vivo murine study after treatment with sodium thiosulphate without significant adverse effects [43]. These results were tested as a single treatment and in combination therapy with Minoxidil. They supported the additional effect of sulphate in Minoxidil therapy due to the cysteine supply. Minoxidil bio-activation by sulfotransferase enzymes has also been highlighted as an important clinical outcome predictor in female hair loss [44]. With the aim of improving topical Minoxidil penetrability, tissue retention and a side effects reduction lecithin-based microparticles has been tested as a vehicle in combination to sulphate [45].

Nowadays, different minoxidil formulations have not showed enough efficacy in all patients and this is the principal cause of the ongoing new therapies research. Specially since there is only indicated treatment for females (topical Minoxidil), nowadays, efforts are being undertaken in order to promote other alternative therapies. Other clinical trials performed with prostaglandin F2 analogues and Cetirizine, that we will address, has the same purpose. AR receptor antagonists outcomes such as from Bicalutamide or Flutamide applications have also been assessed showing mild favourable results in females and hepatotoxicity risk hence its not recomended use [26, 46].

### Prostagladins analogues and antagonists receptors

In general in order to avoid Finasteride and Minoxidil side effects, other topical drugs have been tested (Table 1). Prostaglandin F2 analogues such as Bimatoprost, approved for eyelashes hypotrichosis and Latanoprost, known to induce anagen phase in hair follicles, have been tested. Clinical trials reported are both effective comparing to placebo [47–52].

**Table 1:** Main current therapies in AGA treatment

Therapeutic action	AGA treatment	Route and dose of administration^1^
5-α reductase inhibitors	Finasteride	Oral (1 mg/day)
		Topical
		Microneedling
		Mesotherapy (Minoxidil 0.5%, 1 ml of Finasteride 0.05%, 2 ml of biotin 5 mg/ml and 2 ml of D- panthenol 50 mg/ml)
	Dutasteride	Oral (0.5 mg/day)
		Topical
		Mesotherapy
Vasodilator/Cytoprotective	Minoxidil	Oral (0.5 mg/day)
		Topical
		Mesotherapy (Minoxidil 0.5%, 1 ml of Finasteride 0.05%, 2 ml of biotin 5 mg/ml and 2 ml of D- panthenol 50 mg/ml) / (Minoxidil 5%, amino acids, vitamins, nutritional compounds)
		Microneedling
Antifungal/antisteroid	Ketokenazol	Topical (2% shampoo adjunct to Finasteride)
PG F2 analogue	Bimatoprost	Topical
PG D2 antagonist	Setipiprant	Oral 1000 mg twice daily
H1 Antihistaminic and prostaglandin D2 synthesis reducer	Cetirizine	Topical
AR antagonists	Cortexolone 17a-propionate	Topical solution 5% Twice-daily
	Spironolactone	Oral (25 mg/day)
	Bicalutamide	Oral
		Mesotherapy (1 ml Bicalutamide 0.5%) 3 monthly sessions
	Cyproterone acetate	Oral 2 mg/day
	Flutamide	Topical solution 2% adjuvant to Minoxidil 5%
Wnt pathway activator	SM04554	Topical solution 0.05–0.45%
JAK-STAT pathway inhibitor	JAK inhibitor 1/3	Topical solution 0.46% twice-daily
Physical treatment	Laser therapy	Low-level laser (Light) therapy/1550 nm fractionated erbium laser
Surgical technique	Hair trasplantation	Follicular unit transplantation
Microcirculation enhancement angiogenesis, platelets mobilisation and growth factors, collagen and elastin synthesis	Microneedling	Dermatological roller, nappage and papule injections. Minoxidil 5% weekly, Finasteride 0,05%, FGF
Microcirculation enhancement	Mesotherapy	LC hair essence serum injection (human stem cells, capixyl, hyaluronic acid, and vitamins)
Homogenous distribution of preparations		Minoxidil 0.5%, 1 ml of Finasteride 0.05%, 2 ml of biotin 5 mg/ml and 2 ml of D- panthenol 50 mg/ml)/(Minoxidil 5%, amino acids, vitamins, nutritional compounds)

^1^According to clinical studies cited in the present review

Setipiprant is also an oral prostaglandin D2 (PG D2) receptor antagonist which is overexpressed in AGA patients and related to follicle miniaturization [53]. A clinical trial testing a Setipiprant dose of 2000 mg/day was also conducted with slightly better results in hair density versus control [54]. In order to inhibite PG D2 receptor activation, topical Cetirizine, H1 antihistaminic and PG D2 production reducer, was also tested in a clinical study of 60 subjects. Experimental group showed significantly higher results in hair growth and patient satisfaction than control [55]. Recently Bassiouny et al. (2023) had performed a clinical trial testing topical Cetirizine as a concomitant medication with topical Minoxidil therapy in the treatment of female AGA. Results showed an increase in the hair shaft thickness and a higher clinical improvement. Its anti-inflammatory action can be also responsible of an improvement in AGA conditions [56].

Although prostaglandin F2 analogues are known to cause hair lightening and prostaglandin D2 antagonists to be related to atrophy and alopecia conditions, none of them achieve enough efficacy to be considered as promising therapeutical options.

### Wnt and JAK-STAT regulators

Other pathways such as Wnt and JAK-STAT are known to regulate the hair cycle. Wnt activator and JAK-STAT inhibitor drugs have been tested in the light of in vivo and in vitro experimental studies with favourable results in hair growth [57, 58]. Current clinical trials results are positive for SM04554, a Wnt pathway activator, promoting hair growth and Ki67 expression in the hair bulb [47, 59, 60]. Other such as Valproic acid and Ciclosporine A have also shown an enhancer hair growth effect. Regarding JAK-STAT inhibitor drugs, a JAK inhibitor 1/3 was clinically tested but no significant differences were observed [61].

### Physical treatments

Laser therapy and hair transplant hightlight among physical treatments. In order to stop hair loss in AGA, a wide range of wavelengths has been studied in laser therapy which could stimulate angiogenesis and inflammation possibly mediated by HSP27 leading to follicular stem cell activation [62].

Damage and hair follicle scarring derived from the difficulty in defining adjusted energy parameters are some of the laser therapy limitations [63]. Lower wavelengths are considered as optimal ones around 655 nm [64]. In 2007, Low-Level Laser (Light) Therapy was approved by the FDA as a treatment for hair loss [26, 65, 66]. Several devices using this technology have been applied such as the HairMax Laser Comb (Lexington International LLC, Boca Raton, FL, United States) in 2011 or iRestore Light Therapy Apparatus, tested in a clinical trial in 2015 for male and female AGA [26, 62, 65]. Several randomised, controlled, double-blinded studies results have shown an increased self-rated questionnaire and total hair score in daily and weekly irradiated individuals [67, 68].

Fractionated Erbium laser studies in women with AGA have also reported an improvement in hair density [69–71]. A trial combining 1550 nm fractionated Erbium laser treatment with topical Minoxidil 5% compared to Minoxidil treatment was conducted in 2019. Combined treatment results were significantly higher than Minoxidil alone [72]. Another study tested 1550 nm fractionated Erbium laser and PRP alone and in combination. A synergistic effect was showed in the combinated therapy with the greatest outcome [73].

As European guidelines report, laser can be applied as an ancillary therapy with Finasteride or Minoxidil therefore single laser therapy outcomes does not show efficacy by itself.

Hair transplantation as a surgical technique is based on the sensitivity and distribution of the ARs in different scalp areas. Thus, follicles are extracted from a donor area which is not sensitive to androgen action and then are inserted into the affected scalp. In general, transplantation involves a lasting impact resulting in natural hair growth after 6 months and in a patient's self-esteem significant improvement. Low density of the donor area [74], reduced viability of the cells obtained [75] or curly hair are limitations in the process [76]. Transplantation success depends on the technique, surgeon`s skills and individual characteristics [77]. Follicular unit transplantation is the gold standard technique with a low frequency of complications [26, 78] however AGA progression continues so adjuvant therapies are necessary hence its use in combination to oral Finasteride [1, 74]. One of its limitations is that it provides a partial solution since the other nontransplanted follicles, which remains in the frontal and vertex area, still are contingent to DHT atrophic action.

Apparently Finasteride should show a 100% of successfull outcomes because it acts exactly in the etiologic target. However this is not a fact. Althought DHT production will stop after blocking 5-α reductase by antiandrogen therapies, minituarization process would have been triggered, afecting numerous hair follicles becoming fibrotic stellae. It is seemed that this phenomena will be mostly irreversible although DHT levels were low as clinical evidence shows. Other injury factors such as inflammation or fibrosis are needed to be treated as well. These are the reasons why new regenerative therapies are necessary in order to restore damaged follicular mechanisms caused by a prolonged DHT action. The angiogenic and anti-inflammatory growth factors release, PRP functions and the supply of different cell populations have shown to be promising therapies in a near future.

### Microneedling

On one hand, microneedling is a widely used technique in dermatology in which a large number of microneedles positioned on a dermatological roller on the skin activates the healing process and thus triggers angiogenesis, platelets mobilisation and growth factors, collagen and elastin synthesis [79]. The mechanism of action is based on the aforementioned regenerative activation, the BSCs stimulation caused by scarring, and growth-related genes overexpression such as Vascular Endothelial Growth Factor (VEGF), β-catenin or Wnt pathway products [80]. Hundred cases of mild to moderate AGA were recruited and it was observed that Minoxidil microneedling was more effective than Minoxidil alone. Since then, numerous trials have supported the microneedling sessions efficacy as an adjuvant technique to pharmacological therapy with Minoxidil or Finasteride and after PRP and PRF administration procedure. In 2018, Kumar et al. [81] compared weekly microneedling plus twice-daily application of topical Minoxidil to Minoxidil-treated group. Hair density increased and patient satisfaction score was higher in the former group, although response was not macroscopically significant. Later these results were supported by Bao et al. [82]. Yu et al. [83] also tested an AGA treatment based on a topical fibroblast growth factor (FGF) solution sprayed before microneedling and topical Minoxidil. They observed in this group the most satisfactory results comparing to Minoxidil, FGF or saline alone.

### Mesotherapy

On the other hand, mesotherapy is an intradermal technique which consists of administering pharmacological substances and natural active ingredients diluted in small doses at specific points on the affected scalp. Melo et al. [84] reported a case whose results showed a considerable increase in hair density after 20 treatment sessions using a mixture composed of 1 ml of Minoxidil 0.5%, 1 ml of Finasteride 0.05%, 2 ml of biotin 5 mg/ml and 2 ml of D-panthenol 50 mg/ml. Mesotherapy with natural compounds seemed to be clinically effective as an adjunctive treatment to Minoxidil and Finasteride. Although it is a minimally invasive technique adverse events may include burning, erythema, headaches, subcutaneous necrosis, scalp abscesses and edema [85].

Gajjar et al. [86] conducted a clinical trial to evaluate safety and efficacy of an amino acids, vitamins and other nutritional compounds solution, and compared it to the group treated with topical Minoxidil 5%. They observed no significant differences between groups. Recently Nassar et al. [87] compared LC hair essence serum (formulated with hyaluronic acid, stem cell extract peptides, and zinc arginine and red clover extract) and botulinum toxin A administration. Better results were obtained in LC treated group than in botox treated one although both showed a significant improvement in hair growth.

Jung et al. [88] obtained favourable results in a pilot animal study whereby botox was subcutaneously administered in a stress model mice, and Zhou et al. [89] showed promising results with an favourable safety and efficacy profiles alone and in combination with Finasteride in a clinical study of 63 patients. Aforementioned, Nassar et al. [87] also obtained significant results. Currently a clinical trial is recruiting male and female participants in order to assess its effect in mild to moderate AGA subjects [90]. It has also been studied as a preventive drug by intramuscular injection for the progressive hair loss in AGA men [91].

Simultaneously microcirculation is enhanced by both methods and consequently additional benefits, besides the active ingredient of the solution applied, are provided to the hair growth and a faster and more effective absortion as well. The needles damage along the surface promotes the activation of skin regenerative mechanisms along with angiogenesis.

## Emerging therapies

The number of innovative therapies increases constantly improving or incorporating new physical techniques and active pharmaceutical or living beings extracts ingredients. Among them the most of AGA emerging therapies are regenerative-based whose main favourable actions are angiogenesis activation, growth factors supply and antiinflamatory action (Table 2).

**Table 2:** Main new therapies in AGA clinical treatment

Therapeutic action	AGA treatment	Dose and schedule of clinical administration^1^
Regenerative^3^	Platelet-rich plasma	1.5·10^6^ platelets/µl.monthly for 3 to 6 times
Regenerative^4^	Platelet-rich fibrin	1 ml administered in 4 sessions of 2 weekly intervals
Regenerative^2^	Autologous MSCs	Obtained by Rigenera System from occipital region or extracted from lower abdomen by digestion with 0.075% collagenase
Regenerative^2^	Autologous SVF or enriched with ADRCs	SVF extracted by Puregraft System enriched with 1,000,000 ADRCs or 500,000 ADRCs
		SVF extracted from lower abdomen by collagenase protocol
Regenerative^2^	ASCs-CM	﻿4 treatment sessions every 3–5 weeks
Microcirculation enhancement	Hydradermabrasion	Venus Glow hydradermabrasion device: 4 sessions of treatment at 1-week interval for first 4 weeks 3 sessions of treatment each 4 weeks apart
		Hydrafacial treatments every 4 weeks in combination with daily application of Keravive Peptide Spray
Relaxation effect, blood flow enhancement	Neurotoxines (Botilinum toxin A)	100 U every 3 months for a total of 4 times
Effective active ingredient delivery and retention	Nanostructures (PGA/PGA- Chitosan Hydrogel /Polymeric/ Molybdenum nanoparticles	Experimental phase
	Nanostructured lipid carriers	
	Nanotransferosomes /Gel nanotransferosomes)	
Hair follicle repopulation	Bioinks, bionanomaterials through 3D bioprinting technique	Experimental phase

^1^According to clinical studies cited in the present review

^2^Angiogenic, inmunomodulatory and growth factors release

^3^Oxigenation, vasoconstriction and growth factors release

^4^Retention of small biomolecules and longer growth factors release

### Phytomedicine

Traditionally phytomedicine has been applied in AGA treatment for many years. Topical and subcutaneous administration of plant extracts has been extended in experimental studies showing a favourable hair promoting effect [92, 93].

Nowadays some clinical trials are been performed in order to validate these preliminar results. Among them, niacin, ascorbic acid, vitamin B complex, tocopherol, grape seed, rosemary oil, sage, nettles and *Hibiscus rosasinensis* are used because of their capacity to improve blood supply [94]*. Serenoa repens* extract prevents TGF-β induction, caused by DHT, and interacts with mithocondrial signaling pathway contributing to its protective action [92]. Other are antioxidants which can act against microinflammation (grape seed) or actively inhibits 5α-reductase (green tea [95], ginkgo biloba [94], gingenoside ro [93] or curcumin [96]). Other tea extracts such as Chinese black tea has shown a higher affinity to estrogen receptors promoting also a hair growth enhancer effect [97].

### Neurotoxines

In the last years the study of neurotoxines in disorders like AGA has been documented.

The mechanism of action of Botulinum toxin A particularly is based in its relaxation effect. It is known that a turgency loss can enhance hair growth. Hair follicle is considered as a mechanosensitive organ which can be affected by an increase of occipitofrontal muscle tightening which reduce blood flow. This fact is considered as a hair loss promoter but not a trigger itself [87]. Another suggested Botulinum toxin A function is the inhibition of TGF-β1 released by hair follicles and related to AGA fibrosis and which is considered as a supressor factor of the follicular keratinocyte growth [98].

Specially Botulinum toxin application has recently been considered as an effective and safe therapeutical option for AGA treatment which improves Finasteride and Finasteride plus Minoxidil outcomes as a supplement of these standard therapies [99].

All the clinical trials performed have reported an increase of hair count, clinical response or patient satisfaction [98–102]. Today further neurotoxins implications in AGA clinical improvement are needed to dilucidate. The heterogenous methodology applied and the absence of control studies are some of the weakest points to be improved [103].

### Nanotechnology

Nanotechnology offers innovative solutions in several areas of biomedicine. They have been widely used in wound healing, tissue regeneration, drug delivery systems and personalised medicine [104–106].

Its use in AGA is growing rapidly and it is establishing itself as a promising new therapeutic option. Diverse nanosystems including nanoparticles, nanostructured lipid carriers and nanotransferosomes have been proposed for the treatment of hair follicle disorders.

These systems share a common strategy: achieving a more precise control over drug release and enhancing the efficacy of drug delivery to the target niche as biocompatible complexes. Their size and design facilitate the accumulation of these nanoparticles in follicle casts, effectively serving as drug reservoirs, thereby increasing local drug concentration at the target site while minimising systemic side effects [107].

The topical use of Minoxidil using lecithin based nanoestructures has been used to enhance a percutaneous delivery and to avoid skin side effects showing yielding comparable efficacy with a reduced incidence of skin issues [45]. Polymeric nanoparticles has also been explored as carriers of topical Finasteride [108]. Whereas the reported lecithin based nanoparticles benefits were associated with an enhanced safety profile, the polymeric Finasteride carriers were found to yield a prolongued Finasteride release thereby increasing its time of residence onto the skin [108, 109].

Other nanoparticles conformed by Molybdenum has been also applied. Molybdelum inhibits oxidative stress due to the presence in its composition of transition metal elements with rapid electron transfer. It is suggested to be a promising therapeutic approach alone and in combination with Minoxidil [110]*.*

Additionally, other formulations based on nano-transferosomes, widely used as drug nanocarriers across the skin [111], were also applied for Finasteride administration as a gel form [28].

A recent study has illustrated an increased efficacy of Aminexil, a keratin fibers stimulator and hair growth promoter, loaded in a nanostructured lipid carrier (NLC) in chemotherapy-induced alopecia rats showing an increase in hair growth promotion compared to the use of the commercial product alone [112].

In the context of phytomedicine, green nanomaterials such as Poly-γ-glutamic acid (PGA) nanoparticles have been widely used as delivery agents due to a high biocompatibility and a good safety profile [113]. In particular it has been shown to be an excellent carrier for an herbal mixture consisting of *Phellinus linteus*, *Cordyceps militaris*, *Polygonum multiflorum*, *Ficus carica,* and *Cocos nucifera* oil. PGA as a vehicle of the herbal mixture also promoted a higher hair length, an earlier anagen initiation and a more prolonged anagen phase in C57BL/6N mice. An increase in β-catenin protein expression, a stimulator of the anagen phase, was reported to improve the effect of the herbal mixture alone [113]. This herbal extract has been also carried by PGA in combination to Chitosan Hydrogel supporting these results and reporting an induction of changes in DPCs to a polygonal shape which is associated with an enlargement of the hair bulbs [114].

PGA has also been proven as a curcumic-zinc framework carrier through a microneedle patch with promising results in hair growth [115]. Therefore, nanoparticles offer an innovative approach for treating AGA, through a targeted delivery, which could potentially improve hair growth outcomes. Nevertheless, further research in this area is needed.

### Hydradermabrasion

Other therapies such as hydradermabrasion, an extended method in the aesthetic field, is being clinically assessed for improving AGA outcomes and for enhancing hair quality through Hydraderm and Hydrafacial in combination to Keravive Peptide spray, an hyperconcentrated solution of biomimetic growth factors and dermal proteins. Results are not available yet [116, 117]. This tecnique is also led to enhance microcirculation in affected scalps.

### Regenerative therapies

Regenerative therapies for AGA offer a new perspective, against traditional treatments limitations, with potential long-term solutions and fewer side effects.

These regenerative therapies encompass various alternatives such as PRP and its newly generation of products called PRF, SVF, and stem cell-based therapies, including MSCs condicionated medium (MSCs-CM) and extracellular vesicles application.

Tissue engineering is also gaining ground through the development of new 3D cell structures composed by HFSCs and DPCs embebbed in specific scaffolds which pretend to evolve to functional hair follicles after being transplanted into the bald scalp.

### PRP

Platelet-rich plasma (PRP) represents the main autologous alternative currently utilized in the treatment of AGA by subcutaneous administration. Although it was initially used for connective tissue regeneration in the field of orthopaedics demonstrating its efficacy in varios conditions such as bone breaks [118], ligament tears [119], osteoarthritis [120] and arthritis [121], its application in the AGA field is widespread with a primary function centered on the restoration of the niche environment. Different growth factors contained in alpha granules of platelets, such as VEGF or Plaque Derived Growth Factor (PDGF) stimulate hair regrowth by inducing the activation of genes associated to various biological processes leading to anagen phase start and proliferation, elastin and collagen synthesis and to an extracellular matrix development [122]. Additionally, its effects includes hypoxia reduction, vasoconstriction and inflammation in bald areas while promoting neoangiogenesis [123–125].

In 2014, Khatu et al. [126] conducted a clinical study on 11 AGA non-responders to treatment with Minoxidil or Finasteride patients for 6 months. Each dose was injected twice a month in 4 sessions. Results were evaluated after 3 months macroscopically based on clinical criteria and photography, hair pull test and satisfaction questionnaire. A significant hair density increase was observed.

In 2018, a prospective and comparative study in AGA young men obtained favourable results with an improvement of hair growth in 16 out of 20 participants [127] and in 2019 another prospective and comparative study between PRP and Minoxidil was also conducted [128]. Both groups were treated for 6 months. Standardised tests data, satisfaction surveys and correlation index between platelet concentration and clinical improvement were collected. It was concluded that PRP treatment was more effective than Minoxidil and that platelet count was proportional to hair density increase. The same year a clinical trial with 19 patients, in which PRP plasma was administered every 4 months in a total of 3 times, reported an increase in the number of hair follicles before the second session [129].

Later Pakhomova & Smirnova [130] tested PRP and Minoxidil combination obtaining promising results in male subjects. Recently Qu et al. [131] have proven the therapeutic PRP effect monthly administrated in a total of 3 times in 32 men. Results revealed that PRP treatment produced a significant increase in hair density, hair diameter and anagen hair ratio at month 6 compared to control.

PRP administration has also shown efficacy in female AGA although it is less effective than Minoxidil [132, 133].

### PRF

In spite of the extensive use of PRP, in the management of AGA its use presents important limitations. There is not an established protocol for PRP preparation and effectiveness varies due to different preparation methods so the optimal concentration of platelets, the relative centrifugal force and the possible benefits or not of the presence of leukocytes in the final composition of the PRP remains unknown. In addition, there is a restricted long term efficacy due to the relatively short half-lives of growth factors and prompt release following PRP activation.

Therefore, in an attempt to overcome these limitations, second-generation platelet concentrates, called platelet-rich fibrin (PRF), was developed. PRF is similar to PRP except that PRF naturally contains fibrin for clot scaffolding which allows the retention of small biomolecules, stem cells and high concentrations of host immune cells contributing to tissue healing and regeneration. Its formulation is entirely autologous since anticoagulants are no needed in the preparation and present a much longer release of growth factors due to its 3D scaffold structure [134].

Since its gel-based consistency nature limited its application, in 2014 an injectable generation of PRF was developed. This new injectable formulation obtained by reduction of the speed centrifugation based on the low speed centrifugation concept [135] presented the advantage of use a liquid form before being converted to a fibrin matrix (clot), which allowed a slower and more gradual release of the growth factors [136]. Also an increase release of growth factors when compared to traditional PRF was observed [137].

Numerous studies have investigated the clinical applications of PRF in different regenerative fields including odontology [136], surgery [138], traumatology [139], wound healing [140], facial esthetics [141] and in hair regrowth [142–145].

Different comparative studies have reported PRF to be more effective in improving fat grafting than PRP [141], when fat graft was combined with either PRP or PRF during facial lipostructure surgery or in the treatment of acne scars [146]*.*

In regard to hair regrowth, there is a growing interest in the application of PRF. In 2021, a study conducted by Lu et al. indicated the role of PRF in promoting hair follicule regeneration through the enhancement of cell proliferation, migration and trichogenic inductivity [142]*.*

In addition, recent clinical studies have highlighted the beneficial effect of PRF in the treatment of AGA. Arora et al. (2019), including three patients between 35 and 40 years of age with a varying degree of hair loss, reported an increase in hair density when treated with injectable PRF [143]. In another study conducted by Shashank et al. (2020), a 34-year-old male with hair thinning diagnosed with grade 4, experimented an increase in hair density after PRF sessions [147] and Bhoite et al. (2022) reported a clinically noticeable improvement in the hair growth in 11 out of 15 patients after receiving PRF and microneedling treatment for 4 sessions along with Minoxidil, Finasteride and multivitamin supplements [144].

To date, the largest human clinical trial (168 patients) was conducted by Schiavone et al. reporting a clinical improvement of AGA parameters at month 6 in all the participants after platelet concentrates administration sessions [145].

Currently, PRF therapy represents an effective, safe and inexpensive innovative treatment for AGA. Neverheless further investigation is required in order to optimize preparation protocols for a more effective composition.

### HFSCs, DPCs and MSCs and their derivatives

MSCs has been widely used in the field of regenerative medicine and their secretoma. Derivative products such as MSCs condicionated medium (MSC-CM) and extracellular vesicles are progressively being more investigated in different pathologies. Both MSCs-CM and exosomes (nanomembranous vesicles) contain different biomolecules capable of restoring physiological conditions.

Since most of the beneficial effects associated with the utilization of MSCs arise from their paracrine action, facilitated by different components present in their secretome such as growth factors, cytokines or chemokines, there is a growing interest in investigating MSCs-CM and exosomes. Extracellular vesicles are secreted through paracrine signaling including microRNAs, mRNAs, metabolites, second messengers, adhesion proteins, growth factor receptors, ligands and long-coding RNAs [148]. Among these components, RNA and proteins are the functional ones implicated in tissue regeneration [149].

The effectiveness of MSCs derivatives treatment is atributed to their main function: organs and tissues homeostasis. Additionally, they exhibit the ability to secrete growth factors and anti-inflammatory cytokines thereby participating in inmunomodulation within the niche and in lymphocyte infiltration reduction generally observed in these patients [150, 151]. Although MSCs are found in different anatomical locations such as periosteum, bone trabeculae, synovial membrane, muscle tissue, dermis and bone marrow [152, 153]; it is from adipose tissue where extraction is less complex through a non-invasive process with a high cell yield [154].

For this reason, adipose-derived stem cells (ASCs) are being widely used in the dermatological field, particularly in tissue regeneration, psoriasis and alopecia. In AGA treatment, SVF, derived from adipose tissue, and stem cells (SCs) or their derivative products have demonstrated significant improvement in hair density and diameter according to the lastest reported outcomes.

The most of the publications are refered to its conditioned medium as a growth factors enriched secretome produced by MSCs metabolism but also to SVF as an easily extractable heterogenous adipose cell population composed of adipocytes, preadipocytes, adipose stem cells, endotelial progenitor cells, hematopoietic progenitors, monocytes, leukocytes and pericytes [155–157].

Currently, HFSCs and MSCs derivatives administration is considered as the main focus in numerous lines of hair regrowth research.

### Experimental regeneration based-studies

In 2019 Gentile et al. [158] collected every hair follicle cell function, interactions among them as well as MSCs signalling impact at follicular level, extracted from in vitro experiments to date. These experiments have elucidated the mechanisms which are involved after supplying MSCs to the hair follicle and about their role as intrinsic populations.

In this context ASCs-CM is known to specifically trigger DPCs replication and hair shafts lengthening in isolated hair follicles [159]. Park et al. [155] evaluated enriched medium effects. ASCs-CM promoted DPCs differentiation and epidermal keratinocytes. They differentiated between normoxic and hypoxic conditions and unexpestedly, after 100 µl injection in each group, faster hair activation was detected in the latter.

Studies with ASCs in murids were applied by intradermal administration in the order of 10^6^ cells. Positive results were obtained in 7-week-old C3H/HeN mice after 12 weeks in the 1.5· 10^6^ ASCs intradermally administered and in the 1 ml of ASCs-CM topically administered groups [150]. Results suggested that ASCs and ASCs-CM promote hair growth by increasing DPCs through cell cycle modulation and anagen phase activation. In 2011, Festa et al. [160] indicated that preadipocytes played an important role in hair growth by activating the SCs follicular activity, whereas mature adipocytes did not show this capacity. They administered SVF and isolated adipogenic precursors in 7-week-old mice. This study confirmed the importance of ASCs. Hair growth was observed when isolated adipocyte precursors were administered while no such effect occurred when SVF was applied. Experimental results indicate that mature adipocytes are not the primary adipogenic cell type involved in the induction of stem cell activity in hair follicles and that adipocyte precursor cells are essential for skin epithelial stem cells activation.

Results obtained by He et al. [161] also supported this conclusion. They evaluated the puripotency of CD34 + , CD34- and SVF cells from adipose tissue in a nude mouse model. Results showed that CD34 + cells administration resulted in a higher number of hair follicles than CD34- and SVF groups. These progenitor cells would participate in hair morphogenesis by integrating into the dermal sheath. On the other hand, differentiation to blood vessel endothelial cells was observed in CD34 + and SVF cells. The former group was shown to have a high differentiation potential in skin development.

These studies were supported by other ones based on tissue regeneration such as the one performed by Zografou et al. [162] in which 10^6^ASCs, distributed in 10 spots, were administered in diabetic Spargue Dawley rats. They observed a survival and angiogenesis increase in the grafted areas. In other pathologies such as psoriasis Rokunohe et al. [163] and Lee et al. [164] applied 3·10^6^ ASCs and 4·10^6^ human umbilical cord-derived mesenchymal stem cells (hUCB-MSCs) respectively in the dorsal area of diseased mice and obtained favourable results in terms of expected immunosuppressive activity.

In regard to the use of exosomes in AGA although most of the investigation is still in a preclinical setting. Several case reports has shown promising results. Instead of MSC-CM use, exosomes are more resistant to degradation, citokines and growth factors half-lives are larger and according to Wu et al. (2021) exosomes show a safer and more efficient profile [165]. In hair regeneration preclinical studies, different sources of exosomes have been explored including exosomes derived from dermal papilla cells (DPCs) [166], from adipose-derived stem cells [165], from hair outer root sheath cells [167] or immune cell-derived exosomes such as macrophage extracellular vesicles [168]. In fact perifollicular macrophages are known to activate DPCs promoting anagen phase.

Commonly, results show an enhancement of hair follicle proliferation and migration, an increase of β-catenin expression via Wnt pathway and an aceleration of the anagen onset [168]. In addition exosomes reduce proinflammatory levels [169] and accelerate re-ephitelialization [170].

### Clinical regeneration based-studies

Cell therapy in AGA has been applied by different procedures such as subcutaneous or intradermal administration from different types of sources in the human body, including, autologous HFSCs [171], ASCs extracted from the occipital region for hair transplantation [172] or obtained from the abdominal area, as part of autologous SVF [173].

In regard to ASCs-CM, Fukuoka and Suga [156] proved its efficacy in a clinical trial involving 22 patients, resulting in a significant increase in hair count after treatment in both male and female subjets. Positive results in hair density and thickness parameters were also observed by Shin et al. in a female cohort [174]. At present, a study including 37 participants using two different ASCs-CM concentrations has been completed with pending results [175].

The efficacy of ASCs-CM was further evaluated in a study with 38 participants over 16 weeks, showing a significant increase in hair count and thickness compared to placebo within 8 weeks of application [176]. Moreover, it was also tested female hair loss with the Hair Stimulating Complex, a derivative solution of ASCs-CM enriched with growth factors [177]. The use of HUCB-MSCs conditioned medium with paracrine factors in a experimental solution called NGF-574H was tested by a twice a day topical administration [178]. In addition, small-scale clinical studies were conducted using SVF [179, 180]. A recent clinical trial comparing PRP versus mesotherapy containing the ASCs-CM and a mixture of recombinant growth factors in 100 participans is still awaiting results [181].

ASCs-CM has also been applied in transplantation areas [172] and formulated with several growth factors, interleukin 6 as AAPE Prostemics commercial product via microneedling administration [174, 182].

Zanzottera et al. [172], in order to optimized the SVF extraction process, used the Rigenera® system to obtain a heterogeneous solution of the hypodermis with autologous SCs from the donor occipital area during hair transplantation procedure. The suspension was applied to the scalp areas undergoing hair transplantation in 3 AGA subjects. Monthly follow-up revealed faster healing after transplantation and improved hair growth after two months. Subsequently Gentile et al. [183] isolated HFSCs using Rigenera® Securdrill resulting in a 29% higher density in the treatment area compared to placebo.

Other researchers have suggested that autologous ASCs-enriched fat grafting could be a promising alternative for treating AGA. Hamed Kadry et al. [173] conducted a study comparing PRP and intradermal SVF treatment, showing that SVF-treated patients exhibited more marked improvements in hair count and hair thickness in both sexes. Both treatments has also been compared in a clinical trial of 22 participants [184]. Ghazally et al. also compared PRP and ASCs suspension vs PRP application in the recipient site during follicular unit extraction [185].

On the other hand, Stevens et al. [186] tested a combined treatment of PRP and SVF, resulting in a significant increase of hair density at 6 and 12 weeks after a single injection in 10 AGA subjects. In this context, safety and efficacy of using a biocellular mixture consisting of emulsified adipose-derived tissue SVF and high density PRP concentrate group is under evaluation in comparison to other groups in 60 female subjects. Experimental groups includes adipose-derived cell-enriched SVF, SVF, high density PRP concentrate and high density PRP concentrate alone [187].

Recently El-Khalawany et al. [188] conducted a clinical study with a single administration of autologous SVF in 30 patients, with positive results in terms of hair density, hair thickness, global photography and patient satisfaction.

Since 2008, researchers have showed a growing interest exploring the efficacy and safety of ex vivo-cultured, expanded and autologous cells. These isolated cells include dermal cells from the occipital region [189–192] or in combination with epidermal cells [193–199]. Currently, a clinical trial is going using autologous HFSCs extracted from occipital area [200] with a previous similar one performed using isolating and replicating HFSCs from scalp biopsies [201]. Results from both are pending. Elmaadawi et al. [202] used autologous bone marrow mononuclear cells and HFSCs in different groups to treat refractory alopecia areata and AGA. A significant improvement was observed in all treatment groups after the administration of a solution containing a total of 10^5^ cells. Despite their different origins, both therapies exhibited similar safety and efficacy, presenting a higher efficacy in females.

To date, the only clinical trial detailing a clinical dose of ASCs in AGA treatment is the STYLE Transplantation [203]. They conducted a randomized study including 71 subjects in 4 groups, two of which received SCs enriched population from SVF (high dose- 1·106 ASCs/cm^2^ and low dose 0.5· 10^6^ ASCs/cm^2^) while the other received Puregraft fat graft and saline respectively. Fat and enriched SVF were administered in the subdermal layer and at a rate of 0.1 ml/cm^2^ over a total area of 40 cm^2^. Follow-up was performed at weeks 6, 12, 24 and 52 revealing an increase of 16 hairs in hair count at weeks 12, 24 and 52 in the low-dose group compared to baseline. In addition, more participants in this group showed a higher number of positive responses on the hair satisfaction questionnaire at week 24, followed by those who received only SVF. No severe adverse effects were reported.

According to exosomes, one clinical trial is only ongoing in order to assess eficacy and safety of exosomes versus PRP in AGA treatment [204].

### Tissue engineering and 3D bioprinting techniques

Nowadays tissue engineering development is making a good progress but the construction of a functional hair follicle is still a huge challenge due to the complexe mesenchymal-epithelial interactions [205]. Three are the aims which are pursued in the context of tissue regeneration: inductive signals intake, sustitution of damaged cells or onstruction of 3D structures composed by cells on synthetic or collagen matrix [206]**.**

Currently the cutting edge advances are based on bioinks constituted by isolated and expanded autologous HFCs and DPCs in vitro extracted from a follicular unit extraction, the construction of spheroid cultures of both lines together and the incorporation into in a biomaterial scaffold which are contigent upon a modulation signaling [106, 207]. An appropiate design, the use of a biocompatible material and the viability of the cells are essential points in order to achieve complete and functional hair follicles after transplantation.

3D bioprinting techniques, such as the refered one, offers the possibility of manufacturing constructs that mimic a particular tissue architecture, regardless of its complexity, facilitating the hierarchical arrangement of cells within intricate 3D biomaterials while promoting tissue regeneration. As a consequence, nanomaterials can also be integrated, creating complex biological structures with enchanced properties including biocompatibility and regeneration action [106].

Currently, 3D printing techniques have been used for the regeneration of different types of tissues, including skin [208], cartilage [209], vascular networks [210] or organs such a bioprosthetic ovary [211]. The potential use of this technology in clinical settings addressing hair loss like AGA seems promising.

In this context, a recent study using 3D printing technique incorporating magnesium silicate nanomaterials, manufactured a multicellular micropattern constituded by hair follicle cells and a vascular network which leaded to hair regrowth in an AGA immunodeficient mouse model [212].

Additionally, 3D skin equivalents with hair follicle structures and epidermal-papillary-dermal layers has been developed using skin tissue equivalents [213].

Nevertheless to success in AGA using bioprinting techniques it will be neccesary to develop biomaterials capable of mimicking the intricate structure of the hair follicle and its surrounding microenvironment, while being biocompatible, bioactive and non immunogenic. Due to the complex structure of the hair follicle considered as a dynamic miniorgan, an improved bioprinting method is required in order to replicate as similarly as possible the hair biological conformation. The presence of factors to be incorporated to the nanostructure providing to the hair follicle microenvironment the functional activity of the absent sebaceous gland is also a challenge.

## Conclusion

Although topical Minoxidil and oral Finasteride are the only approved drugs for AGA, numerous adverse events are associated with their administration. Hair transplant is an effective option with favourable results, however long-term efficacy may diminish due to progressive miniaturization and loss of preexisting nontransplanted hairs induced by DHT chronic action. The elucidation of strategies to ameliorate the AGA hindered microenvironment is a complex challenge. Recurrent local hormonal action can jeopardize follicle funcionality and regular cycling processes. Early therapeutic intervention is crucial in order to preserve follicles and prevent irreversible damage and fibrosis.

Currently, different strategies aiming to restore physiological conditions or the hair follicle are being under investigation. Allogenic and autologous stem cells administration, along with their derivatives including ASCs-CM or exosomes, are well known for supplying essencial growth factors pivotal for the niche restoration. In addition, their immunomodulatory role plays a crucial role to ameliorate microinflammation associated with AGA and despite of the numerous advantages potentially offered by these therapies their widespread application hinders their implementation. Additionally, to support the clinical use of MSC derivatives, there is a need to standardise and optimise preparation protocols to tackle issues related to donor variability or tissue origin that influence the secretome composition and thereby their therapeutic action.

In addition, contemporary literature highlights research studies on bioinks and bionanomaterial scaffolds for 3D bioprinting techniques across different fields. Despite being in the early stages of exploration, these techniques show considerable potential and offer significant promise for the treatment of AGA. The intricate nature of biological systems, exemplified by the dynamic life cycle of the hair follicle, presents pivotal considerations in the assembly of multi-layered scaffolds. The construction of spheroidal mix cultures of HFSCs and DPCs embebbed in scaffolds has replaced isolated and expanded HFCS administration alone. The DPCs and HFCs interaction is the basis for the onset of anagen phase so an adecuate choice of trigger factors which would induce bioink grafting and transformation into a functional biological struture is essential. The adecuate scaffold biomaterial should be also biocompatible and resilient enough to persist in the dermal layers until progression to become a functional hair follicle analogue structure.

The aforementioned regenerative approaches are leading to two different pursuits. One focuses on the restoration of miniaturized hair follicles and the altered microenviroment surrounding them to rekindle inherent selfrenewal capacities whereas the other one aims to recreate from scratch a structure as complex as such as the hair follicle. It would be necessary to assess patient clinical characteristics carefully according to AGA severity and the onset of alopecic conditions in order to set a treatment which pretends to revive his own follicular regenerative mechanisms or to create de novo lost hair follicles which became fibrotic stellae.

Further investigation is needed in order to define the exogen factors that could lead to functional hair follicle development from bioinks.

## Data Availability

All references are included in this review.

## Abbreviations

AGA: androgenetic alopecia
AR: androgen receptor
ASCs: adipose derived stem cells
ASCs-CM: adipose derived stem cells conditioned medium
DHT: dihydrotestosterone
DPCs: dermal papilla cells
FGF: fibroblast growth factor
HFSCs: hair follicle stem cells
HUCB-MSCs: human umbilical cord blood-derived mesenchymal stem cells
LLLT: low-level laser therapy
MSCs: mesenchimal stem cells
PDGF: plaque derived growth factor
PRP: platelet rich plasma
PRF: platelet rich fibrin
TGF: transforming growth factor
VEGF: vascular endotelial growth factor
